# Comparing the diagnostic accuracy of computed tomography vs transoesophageal echocardiography for infective endocarditis – A meta-analysis

**DOI:** 10.12669/pjms.38.3.5139

**Published:** 2022

**Authors:** Liqin Jing, Yanchun Song

**Affiliations:** 1 Liqin Jing, Department of Ultrasound, Shengli Oilfield Central Hospital, Dongying 257200, Shandong Province, P.R. China; 2 Yanchun Song, Department of Ultrasound, Shengli Oilfield Central Hospital, Dongying 257200, Shandong Province, P.R. China

**Keywords:** Cardiac Computed Tomography, Comparison Review, Diagnostic Performance, Infective Endocarditis, Transesophageal Echocradiography

## Abstract

**Objective:**

To investigate the comparative diagnostic accuracy of cardiac computed tomography (CT) and transoesophageal echocardiography (TEE) for detecting infective endocarditis.

**Methods:**

Original publications published in English language before July, 2021 were thoroughly search in PubMed, CENTRAL (Cochrane Central Register of Controlled Trials), and Google Scholar literature databases. Studies were included if they used CT and/or TEE as an index test, presented data on valvular complications related to infective endocarditis, and used surgical findings as to the reference standard.

**Results:**

Literature screening identified fifteen studies that fulfilled the inclusion criteria. Meta-analysis showed that CT sensitivity for detecting valvular abscesses was higher than that of TEE [0.88 (95% confidence interval [CI]: 0.82 to 0.94; 11 studies involving 842 subjects) versus 0.74 (95%CI: 0.65 to 0.84) P = 0.015; 12 studies involving 917 subjects]. TEE showed statistically significantly greater sensitivity than CT for detecting valvular vegetation [0.91 (95% CI: 0.84 to 0.97, 11 studies involving 971 subjects) versus 0.80 (95% CI: 0.69 to 0.82), 12 studies involving 915 subjects, P =0.019. In case of leaflet detection, TEE showed statistically significantly higher sensitivity than CT (0.76 vs 0.46, P =0.010).

**Conclusion:**

CT performs statistically significantly better than TEE for detecting abscesses while TEE provides statistically significant superior results for detecting vegetation. There is a need for well-designed prospective studies to further corroborate these findings.

## INTRODUCTION

Infective endocarditis (IE) is a devastating health concern typically requiring urgent surgical intervention, especially for patients with signs and symptoms of persistent infection, intractable cardiac failure, severe valvular dysfunction, or perivalvular abscess formation. The mortality rate can be up to 40%, although, in patients with large vegetation, early surgery can reduce mortality and the incidence of systemic embolic events relative to conventional treatment.^1^ pre-operative evaluation of the affected valves is important, especially in terms of functional and anatomical deterioration. This information is necessary to assist surgeons to better plan for surgery and also the timely operations that may be provided to the patients. A study reported from a tertiary care hospital in Pakistan found that infective endocarditis was associated with 32% of inpatient mortality.^2^ Another retrospective study from Pakistan showed 27% of overall mortality.

The gold standard diagnostic method for diagnosing structural abnormalities in IE is transesophageal echocardiography (TEE).^3^ This technique can be easily performed without exposing patients to radiation. Vegetation, ring abscesses, pseudoaneurysms, fistulae, leaflet perforation, and valvular dehiscence are all characteristic IE symptoms identified by TEE. However, definitively diagnosing vegetation and perivalvular extensions throughout the heart is not always easy because of the limited echo window and potential disease complexity. Recent years have seen rapid advances in cardiac computed tomography (CT), which has led to its use for cardiac and coronary artery imaging.^4^ Moreover, advances in terms of temporal and spatial resolution now allow CT scanners to facilitate high-resolution cardiac imaging. As such, over the past decade, CT has been used as an alternative imaging modality for diagnosing IE. That said, CT has several disadvantages, namely that it subjects patients to radiation exposure and that it cannot be conducted at the bedside.

CCT’s diagnostic value is currently limited by a few numbers of investigations, and its effectiveness in comparison to TEE is unknown.^5^ Therefore, we conducted a meta-analysis of existing published studies to compare the diagnostic accuracy of CT and TEE for the diagnosis of IE and its complications.

## METHODS

### Search Strategy

We searched four publicaly available academic databases—PubMed, Scopus, CENTRAL (Cochrane Central Register of Controlled Trials), and Google Scholar—for English language articles published before June 2021. The search was conducted according to PRISMA and Cochrane guidelines.^6^ The following search terms were employed: (“tomography, computed”[MeSH Terms] “echocardiography, transesophageal”[MeSH Terms] OR (“echocardiography”[All Fields] AND (“endocarditis”[MeSH Terms] OR “endocarditis”[All Fields] OR “endocarditis”[All Fields]). We also scanned reference lists of included studies and pertinent review articles to identify additional candidates for inclusion.

### Inclusion criteria:

- As an index test, the researchers performed CT and/or TEE,
- Data on valvular consequences of infective endocarditis (abscess/ pseudoaneurysm, vegetation, leaflet perforation, or fistula) was provided,
- Surgical findings were used as the reference standard. (Studies conducted on patients with both native and artificial valves were included). Case studies, letters, and reviews were not included.

### Data collection and analysis

Inclusion was determined by two independent current authors based on the criteria listed above. Participant details, study methods, interventions, and outcome measurements were extracted from individual studies and summarized for further analysis.

### Statistical analyses

A random-effects model was used to calculate pooled sensitivity/specificity with a 95% confidence interval (CI) in case of heterogeneity of more than 50%, otherwise, a fixed-effect model was applied. Heterogeneity was calculated using the I^2^ statistic. In cases where confidence intervals were not reported, we computed them manually based on the available data. Statistical analyses were conducted by the statistical software STATA (Version 13, College Station, TX: StataCorp LP).

## RESULTS

**Fig.1:**
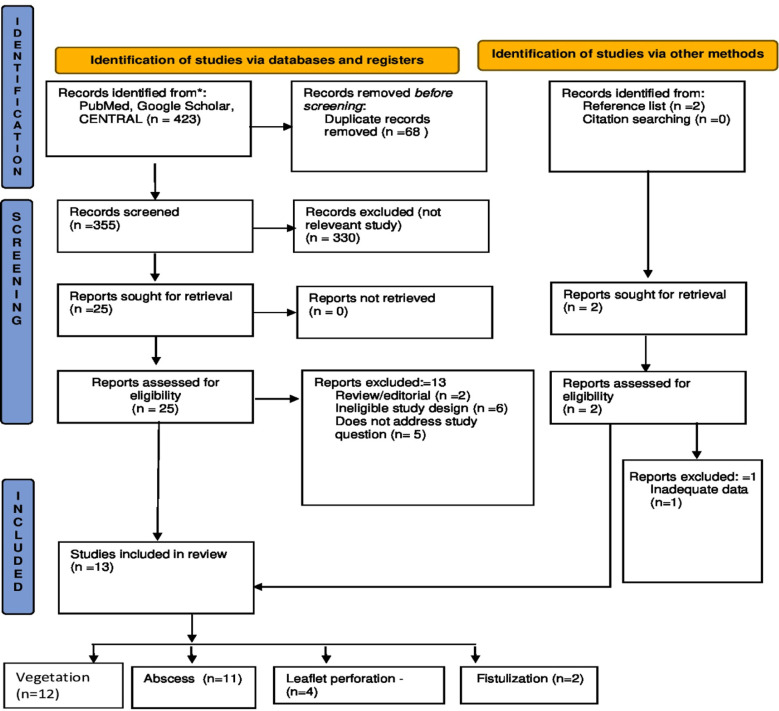
Study inclusion flow diagram.

The initial search of PubMed, Scopus, CENTRAL (Cochrane Central Register of Controlled Trials), and Google scholar databases yielded 423 results. Of these, 423 a total of 13 studies met all inclusion criteria (Fig.1). The fifteen studies included for meta-analysis were published between 2009 and 2020 (Table-I). Study population size ranged from 19 to 251 IE patients. Finally, studies were based in the USA (3 total)^5^,^7^–^9^, Sweden^10^,^11^, France^12^,^13^, South Korea (2 each)^14^,^15^, Austria^16^, Japan^17^, Poland^18^, Thailand^19^, and China (1 each).^20^

**Table I:** Baseline patient information for included studies.

Author; Year; Country; Study design; Sample size	Inclusion Period	Study Population	Age; Mean + SD	Male		Valve involved in disease Process	The interval between TEE and CT	The interval between reference standard and TEE	The interval between the reference standard and CT
Feuchtner et al 2009^15^; Austria; Retrospective; 37	2006-2007	Clinically Suspected IE	56 (20-84)	26	CT & TEE	Native & Prosthetic	1 Days	NR	5 Days
Gahide et al 2010^11^; France; Prospective; 19	2004-2008	Aortic IE requiring Surgery	55 + 13	18	CT	Native & Prosthetic	NR	NR	NR
Fagman et al 2012^9^; Sweden; Prospective; 27	2008-2011	Suspected Arotic IE	68 (24-81)	25	CT & TEE	Prosthetic	5 Days	5 Days	3 Days
Koo et al 2018^13^; South Korea; Retrospective; 49	2011-2013	Patients with surgery for IE	54 + 17	34	CT & TEE	Native & Prosthetic	1.6 + 1.8	NR	2.4 + 1.7
Sims et al 2018^7^; USA; Retrospective; 251	2006-2014	Patients with surgery for IE	54	196	CT & TEE	Native & Prosthetic	NR	1 Days	4 Days
Ouchi et al 2018^16^; Japan; Retrospective; 14	2008-2017	Patients with surgery for IE	66 (28-85)	9	CT & TEE	Native & Prosthetic	NR	NR	NR
Konero et al 2018^4^; USA; Retrospective; 122	2007-2014	Patients with surgery for IE	NR	83	CT & TEE	Native & Prosthetic	2 Days	6 Days	4 Days
Hryniewiecki et al 2019^17^; Poland; Prospective; 53	2011-2015	Patients with IE	58.3 (22-84)	42	CT & TEE	Native & Prosthetic	3.9 + 4.7 Days	NR	8.3 + 12.1
Chaosuwannakit et al 2019^18^; Thailand; Retrospective; 24	2015-2017	Patients with surgery for IE	NR	NR	CT & TEE	Native & Prosthetic	2 Days	7 Days	5 Days
Sifaoui et al 2020^12^; France; Prospective; 68	2015-2017	Patients with surgery for IE	63 + 2	57	CT & TEE	Native & Prosthetic	NR	NR	NR
Kim et al 2018^14^; Korea; Retrospective; 75	2008-2015	Patients with surgery for IE	58.2 + 15	53	CT & TEE	Native & Prosthetic	3 Days	NR	NR
Velangi et al 2020^8^; USA; Retrospective; 73	2010-2018	Patients with IE	62.1 + 16.5	48	CT & TEE	Prosthetic	NR	Within 1 year of reoperation	Within 1 year of reoperation
Ye et al 2020^19^; China; Retrospective; 178	2008-2019	Patients with IE	54 (39-69)	147	CT & TEE	Native & Prosthetic	NR	NR	NR

TEE sensitivity for vegetation detection was superior as compared to CT [0.91 (95% CI: 0.84 to 97), 11 studies involving 971 subjects versus 0.80 (95% CI: 0.69 to 0.82), 12 studies involving 915 subjects, P =0.019] (Table-II). However, no difference between CT specificity and TEE specificity was noted [0.80% (95% CI: 0.62% to 0.94) based on seven studies vs 0.80 (95% CI: 0.71 to 0. 90) based on eight studies (P =0.99).

The pooled sensitivities and specificities for the detection of peri-annular complications using CCT or TEE as shown in Table-II. CT sensitivity for detecting abscesses and pseudoaneurysms was higher than that of TEE [0.88 (95% CI: 0.82, 0.94), 11 studies involving 842 subjects versus 0.74 (95% CI: 0.65%, 0.84) based on 12 studies involving 917 subjects, P =0.015]. However, TEE specificity was statistically non-significantly higher than that for CT [0.89 (95% CI: 0.80 to 0.97), five studies involving 643 subjects versus 0.86 (95% CI: 0.79 to 0.93), five studies involving 643 subjects, P= 0.59].

**Table II:** Comparision of diagnostic accuracy of CCT and TEE.

Characteristics	CCT	TEE	P value
***Vegetation***			
Sensitivity	0.80(0.69 to 0.82)	0.91(0.84 to 0.97)	0.019
	N=12	N=11	
Specificity	0.80(0.71 to 0.90)	0.80(0.62 to 0.94)	1
	N=8	N=7	
***Peri-annular complications (abscesses and pseudoaneurysms)***			
Sensitivity	0.88(0.82 to 0.94)	0.74(0.65 to 0.84)	0.015
	N=11	N=10	
Specificity	0.86(0.79 to 0.93)	0.89(0.80 to 0.97)	0.59
	N=5	N=5	
***Leaflet Perforation***			
Sensitivity	0.46(0.24 to 0.68)	0.76(0.70 to 0.81)	0.010
Specificity	-	0.88(0.76 to 1)	
		N=3	
***Fistula***			
Sensitivity	0.79(0.32 to 1)	0.91(0.73 to 1)	0.52
	N=2	N=2	
Specificity	0.98(0.96 to 1)	0.98(0.97 to 1)	1
	N=2	N=2	

**Fig.2:**
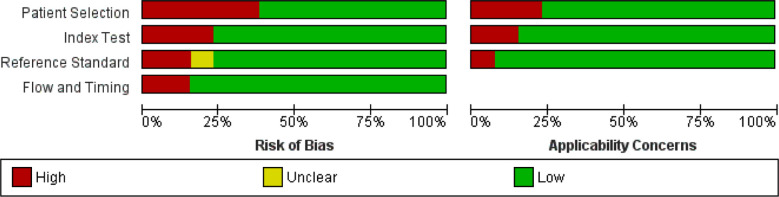
QUADAS-2 score, risk of bias in each individual domain for quality assessment.

We observed evidence that TEE sensitivity was statistically significantly higher for leaflet perforation compared to CT (0.76 vs 0.46, P = 0.010) Table-II Based on three studies specificity for TEE for detecting leaflet perforation was 0.88(95% CI 0.76 to 1), however, we could not compute the specificity for CT due to an insufficient number of studies that reported the data for the same. Only two studies reported the data for fistula detection. We observed a non-significantly higher sensitivity of TEE for detecting fistula compared to CT (0.91 vs 0.79, P =0.52), however, no difference was noted between both for specificity (P=0.99) Table-II. The overall risk of bias was moderate among the studies included in the present meta-analysis (Fig.2).

## DISCUSSION

In this meta-analysis, we compared the diagnostic performance of TEE and CCT in patients with IE. We observed that TEE had superior performance shown by higher sensitivity than CCT for diagnosing vegetation however, CT had statistically significantly higher sensitivity for detecting peri-annular complications.

The current meta-analysis adds to the growing body of evidence supporting the use of CCT and TEE for the correct diagnosis of IE. Historically, TTE has provided reliable diagnostic criteria for first-line examinations and evaluations of IE patients.^21^ However, many patients additionally require further diagnostic options, especially to detect severe complications like peri-valvular abscesses.^22^ Moreover, an additional diagnostic evaluation is often required to facilitate decision-making regarding surgical management.^22^ The invasiveness of TEE limits its use, and patients may have contraindications such as active gastroesophageal bleeding.^22^

Although some recent studies have evaluated the significance of CCT in IE diagnosis, few have directly compared CCT and TEE in the same patient demographics. As a result we combined data from studies investigating only CCT or only TEE to increase the power to detect this association. CT may have the potential to provide improved diagnostic information. For identifying smaller vegetations (less than 10 mm) TEE is more reliable due to higher temporal resolution,^23^ but this observation could not be verified in current study because not all studies provided detailed information on vegetation size.

Overall, our research shows that both imaging modalities are equally relevant and reliable diagnostic modalities for evaluating IE. Our findings support the idea that CCT can be used as a supplement to TEE, especially in cases when TEE is negative or inconclusive but there is a clinical suspicion of IE.^24^ Moreover, if the potential for the complication is high or if TEE is contraindicated, CCT provides a viable alternative.^25^

CCT’s importance has been recognized in the latest endocarditis management guidelines.^21^ When compared to other imaging modalities such as PET and magnetic resonance imaging, CCT is non-invasive and readily available.^26^ As part of preoperative examinations, coronary CT angiography has a high negative predictive value for detecting coronary artery disease.^27^

Our study represents, at present, the large body of evidence supporting a complementary role for CCT and TEE during IE diagnosis. TEE is an effective diagnostic tool for detecting endocarditis and its associated cardiac problems, but CCT offers distinct advantages in cases of prosthetic valve endocarditis and peri-annular complications. Furthermore, CCT is a non-invasive diagnostic technique that aids in surgery planning. All of this suggests that combining the two modalities may be the optimum option. CCT should be investigated in cases when clinical suspicion persists despite a negative or suspicious TEE, as well as suspected uncontrolled infection from endocarditis sequelae that are not detected by TEE. Combined used of CCT and TTE for identifying vegetations and peri-annual problems was studied by Hryniewiecki et al.^18^ which revealed a combined sensitivity of 100 percent. Likewise, a study published by Wang et al. also demonstrated the complementarity for CCT and TTE when applied to decision-making for endocarditis surgery.^28^

### Limitations of Meta Analysis

In particular, CCT was used for diagnosing IE patients with additional risk factors such as prosthetic valve IE, previous cardiac surgery, aortic valve IE, and evaluation of root complications. Many studies also did not report 95% CIs for specificity, as such, there is a possibility that computed 95% CIs may overestimate pooled sensitivity and specificity.

## CONCLUSION

For detecting vegetation and abscesses, CCT and TEE have moderate to high sensitivities and specificities. When compared with each other, CCT was statistically significantly more sensitive for detecting abscesses while TEE was statistically significantly more sensitive for detecting vegetations. The proper application of both TEE and CCT in clinical practice may result in better diagnostic results. Further investigation is needed to validate the findings of this study.

### Authors’ contributions:

**LJ:** Conceived and designed the study.

**LJ and YS:** Collected the data and performed the analysis.

**LJ:** Involved in the Writing of the manuscript and is responsible for integrity of the study.

**YS:** Made significant contribution to the study at different stages besides editing the manuscript.

All authors have read and approved the final manuscript.

## References

[1] Pant S, Patel NJ, Deshmukh A, Golwala H, Patel N, Badheka A (2015). Trends in infective endocarditis incidence, microbiology, and valve replacement in the United States from 2000 to 2011. J Am Coll Cardiol.

[2] Arshad S, Awan S, Bokhari SS, Tariq M (2015). Clinical predictors of mortality in hospitalized patients with infective endocarditis at a tertiary care center in Pakistan. J Pak Med Assoc.

[3] Baddour LM, Wilson WR, Bayer AS, Fowler VG, Tleyjeh IM, Rybak MJ (2015). Infective Endocarditis in Adults:Diagnosis, Antimicrobial Therapy, and Management of Complications:A Scientific Statement for Healthcare Professionals from the American Heart Association. Circulation.

[4] Heseltine TD, Murray SW, Ruzsics B, Fisher M (2020). Latest Advances in Cardiac CT. Eur Cardiol Rev.

[5] Koneru S, Huang SS, Oldan J, Betancor J, Popovic ZB, Rodriguez LL (2018). Role of preoperative cardiac CT in the evaluation of infective endocarditis:comparison with transesophageal echocardiography and surgical findings. Cardiovasc Diagn Ther.

[6] Moher D, Shamseer L, Clarke M, Ghersi D, Liberati A, Petticrew M (2015). Preferred reporting items for systematic review and meta-analysis protocols (PRISMA-P) 2015 statement. Syst Rev.

[7] Wang TKM, Bin Saeedan M, Chan N, Obuchowski NA, Shrestha N, Xu B (2020). Complementary Diagnostic and Prognostic Contributions of Cardiac Computed Tomography for Infective Endocarditis Surgery. Circ Cardiovasc Imaging.

[8] Sims JR, Anavekar NS, Chandrasekaran K, Steckelberg JM, Wilson WR, Gersh BJ (2018). Utility of cardiac computed tomography scanning in the diagnosis and pre-operative evaluation of patients with infective endocarditis. Int J Cardiovasc Imaging.

[9] Velangi PS, Kalra R, Markowitz J, Nijjar PS (2020). Utility of CT in the diagnosis of prosthetic valve abnormalities. J Card Surg.

[10] Fagman E, Perrotta S, Bech-Hanssen O, Flinck A, Lamm C, Olaison L (2012). ECG-gated computed tomography:a new role for patients with suspected aortic prosthetic valve endocarditis. Eur Radiol.

[11] Fagman E, Flinck A, Snygg-Martin U, Olaison L, Bech-Hanssen O, Svensson G (2016). Surgical decision-making in aortic prosthetic valve endocarditis:the influence of electrocardiogram-gated computed tomography. Eur J Cardio-Thorac Surg Off J Eur Assoc Cardio-Thorac Surg.

[12] Gahide G, Bommart S, Demaria R, Sportouch C, Dambia H, Albat B (2010). Preoperative evaluation in aortic endocarditis:findings on cardiac CT. Am J Roentgenol.

[13] Sifaoui I, Oliver L, Tacher V, Fiore A, Lepeule R, Moussafeur A (2020). Diagnostic Performance of Transesophageal Echocardiography and Cardiac Computed Tomography in Infective Endocarditis. J Am Soc Echocardiogr Off Publ Am Soc Echocardiogr.

[14] Koo HJ, Yang DH, Kang J-W, Lee JY, Kim DH, Song JM (2018). Demonstration of infective endocarditis by cardiac CT and transoesophageal echocardiography:comparison with intra-operative findings. Eur Heart J Cardiovasc Imaging.

[15] Kim IC, Chang S, Hong GR, Lee SH, Lee S, Ha JW (2018). Comparison of Cardiac Computed Tomography With Transesophageal Echocardiography for Identifying Vegetation and Intracardiac Complications in Patients With Infective Endocarditis in the Era of 3-Dimensional Images. Circ Cardiovasc Imaging.

[16] Feuchtner GM, Stolzmann P, Dichtl W, Schertler T, Bonatti J, Scheffel H (2009). Multislice computed tomography in infective endocarditis:comparison with transesophageal echocardiography and intraoperative findings. J Am Coll Cardiol.

[17] Ouchi K, Sakuma T, Ojiri H (2018). Cardiac computed tomography as a viable alternative to echocardiography to detect vegetations and perivalvular complications in patients with infective endocarditis. Jpn J Radiol.

[18] Hryniewiecki T, Zatorska K, Abramczuk E, Zakrzewski D, Szymański P, Kuśmierczyk M (2019). The usefulness of cardiac CT in the diagnosis of perivalvular complications in patients with infective endocarditis. Eur Radiol.

[19] Chaosuwannakit N, Makarawate P (2019). Value of cardiac computed tomography angiography in pre-operative assessment of infective endocarditis. J Cardiothorac Surg.

[20] Ye W, Ren G, Zhong X, Jian X, Chen O, Ma Q (2020). ECG-gated CT in Aortic Perivalvular Abscess:Comparison with Transesophageal Echocardiography and Intraoperative Findings. Radiology.

[21] Barton TL, Mottram PM, Stuart RL, Cameron JD, Moir S (2014). Transthoracic echocardiography is still useful in the initial evaluation of patients with suspected infective endocarditis:evaluation of a large cohort at a tertiary referral center. Mayo Clin Proc.

[22] Habib G, Badano L, Tribouilloy C, Vilacosta I, Zamorano JL, Galderisi M (2010). Recommendations for the practice of echocardiography in infective endocarditis. Eur J Echocardiogr J Work Group Echocardiogr Eur Soc Cardiol.

[23] Mugge A, Daniel WG, Frank G, Lichtlen PR (1989). Echocardiography in infective endocarditis:reassessment of prognostic implications of vegetation size determined by the transthoracic and the transesophageal approach. J Am Coll Cardiol.

[24] Sordelli C, Fele N, Mocerino R, Weisz SH, Ascione L, Caso P (2019). Infective Endocarditis:Echocardiographic Imaging and New Imaging Modalities. J Cardiovasc Echography.

[25] Romero J, Husain SA, Kelesidis I, Sanz J, Medina HM, Garcia MJ (2013). Detection of Left Atrial Appendage Thrombus by Cardiac Computed Tomography in Patients With Atrial Fibrillation:A Meta-Analysis. Circ Cardiovasc Imaging.

[26] Mordi IR, Badar AA, Irving RJ, Weir-McCall JR, Houston JG, Lang CC (2017). Efficacy of noninvasive cardiac imaging tests in diagnosis and management of stable coronary artery disease. Vasc Health Risk Manag.

[27] Coronary computed tomography angiography for risk stratification before noncardiac surgery (2016). Ann Card Anaesth.

[28] Wang A,Gaca JG, Chu VH (2018). Management Considerations in Infective Endocarditis:A Review. JAMA.

